# Monitoring of patients treated with lithium for bipolar disorder: an international survey

**DOI:** 10.1186/s40345-018-0120-1

**Published:** 2018-04-14

**Authors:** M. Nederlof, E. R. Heerdink, A. C. G. Egberts, I. Wilting, L. J. Stoker, R. Hoekstra, R. W. Kupka

**Affiliations:** 1 Division of Pharmacoepidemiology and Clinical Pharmacology, Utrecht Institute for Pharmaceutical Sciences, Utrecht University, 3508 TB Utrecht, The Netherlands; 2 Brocacef Ziekenhuisfarmacie, 3600 AB Maarssen, The Netherlands; 3 Research Group Innovation of Pharmaceutical Care, University of Applied Sciences Utrecht, 3508 AD Utrecht, The Netherlands; 4 Department of Clinical Pharmacy, University Medical Center Utrecht, 3508 GA Utrecht, The Netherlands; 5 Antes, Delta Psychiatric Center, 3709 DZ Rotterdam, The Netherlands; 6 Department of Psychiatry, Amsterdam Public Health Research Institute, VU University Medical Center, A.J. Ernststraat 1187, 1081 HL Amsterdam, The Netherlands

**Keywords:** Lithium, Bipolar disorder, Survey, Monitoring, Therapeutic drug monitoring

## Abstract

**Background:**

Adequate monitoring of patients using lithium is needed for optimal dosing and for early identification of patients with (potential) ADEs. The objective was to internationally assess how health care professionals monitor patients treated with lithium for bipolar disorder.

**Methods:**

Using networks of various professional organizations, an anonymous online survey was conducted among health care professionals prescribing lithium. Target lithium serum levels and frequency of monitoring was assessed together with monitoring of physical and laboratory parameters. Reasons to and not to monitor and use of guidelines and institutional protocols, and local monitoring systems were investigated.

**Results:**

The survey was completed by 117 health care professionals incorporating responses from twenty-four countries. All prescribers reported to monitor lithium serum levels on a regular basis, with varying target ranges. Almost all (> 97%) monitored thyroid and renal function before start and during maintenance treatment. Reported monitoring of other laboratory and physical parameters was variable. The majority of respondents (74%) used guidelines or institutional protocols for monitoring. In general, the prescriber was responsible for monitoring, had to request every monitoring parameter separately and only a minority of patients was automatically invited.

**Conclusions:**

Lithium serum levels, renal and thyroid function were monitored by (almost) all physicians. However, there was considerable variation in other monitoring parameters. Our results help to understand why prescribers of lithium monitor patients and what their main reasons are not to monitor patients using lithium.

**Electronic supplementary material:**

The online version of this article (10.1186/s40345-018-0120-1) contains supplementary material, which is available to authorized users.

## Background

Mood stabilizers play a pivotal role in the long-term treatment of patients with bipolar disorder. While it is known that mood stabilizers effectively treat symptoms of bipolar disorder, the potential for adverse drug events (ADEs) is of concern (Rothschild et al. 2007; Ayani et al. 2016; Mann et al. 2008). Some ADEs are preventable, and steps to minimize errors are needed to improve patient safety (Leendertse et al. 2008).

Lithium is the gold standard for maintenance treatment of patients with bipolar disorder (Sani et al. 2017). It is a drug with a narrow therapeutic index; therefore, careful therapeutic drug monitoring is needed to maximize effectiveness and to minimize ADEs and toxicity. Patients using lithium are known to have a high intra- and interpatient variability in dose—concentration relationship, and external factors including drug–drug interactions, environmental temperature, and fluid and electrolyte intake may influence lithium serum levels (Amdisen 1980; Huang et al. 2008; Wilting et al. 2005; Wilting et al. 2007; Rej et al. 2014). Recommendations regarding optimal lithium serum levels differ among clinical practice guidelines, with the most common range being between 0.6 and 0.8 mmol/L for maintenance treatment (Malhi et al. 2017). The illness stage may further require a different approach for lithium serum levels (Malhi et al. 2016).

Besides monitoring of lithium serum levels, clinical and biomarker monitoring of physical and laboratory parameters is required (Nederlof et al. 2015). Lithium use has been associated with a gradual decline in renal function: estimated glomerular filtration rate (eGFR) decreases by about 30% more than associated with aging alone (Tondo et al. 2017). This decline in renal function can become irreversible and may lead to renal failure (Gitlin 2016). Moreover, a decline in eGFR can lead to an increased risk of lithium toxicity due to accumulation (Lepkifker et al. 2004). Furthermore, lithium is known to affect thyroid function (van Melick et al. 2010); with up to 40% of patients experiencing goiter and about 20% hypothyroidism (Lazarus 2009), although the reported prevalence shows a wide range. A 10% higher value of parathyroid hormone (PTH) and calcium was found in a meta-analysis comparing patients using lithium to healthy controls or psychiatric patients not treated with lithium (McKnight et al. 2012). These elevations could stimulate pathological changes leading to the occurrence of hyperplasia or parathyroid adenomas (Giusti et al. 2012). In addition, physical parameters such as weight and blood pressure may be influenced during lithium use (Gitlin 2016; Bisogni et al. 2016).

Adequate monitoring of patients during lithium use is needed for early identification of patients with (potential) ADEs and for optimal dosing (Kirkham et al. 2013; Severus et al. 2008; Aral and Vecchio-Sadus 2008; Baird-Gunning et al. 2017). So far, it is not known how different health care professionals internationally aim to achieve safe and effective lithium treatment through monitoring. Local monitoring systems may differ and health care professionals may be influenced by different guidelines, institutional or laboratory protocols, scientific literature, and their own personal knowledge and experience. The objective of this study was therefore to internationally assess how health care professionals report to monitor patients treated with lithium.

## Methods

### Study design, participants and recruitment

An international survey was conducted between October 2016 and April 2017 among health care professionals prescribing lithium for patients with bipolar disorder. Health care professionals were recruited through networks of various professional organizations (Additional file 1: Appendix S1), and invited by email to participate. The email introduced the nature and purpose of the survey and proposed to further forward the survey to lithium prescribing colleagues. To maximize the response rate, attention was generated during presentations for the Dutch Foundation for Bipolar Disorders and the Belgian College of Neurological and Biological Psychiatry and through flyers at a meeting of the International Group for The Study of Lithium Treated Patients (IGSLi). The survey was conducted in English. Participation was voluntary and answers were kept with anonymized identity of participants. A reminder email was sent a few weeks following the initial request. Study data were analyzed at the Division of Pharmacoepidemiology and Clinical Pharmacology of Utrecht University. The study protocol was approved by the Institutional Review Board of the Division of Pharmacoepidemiology and Clinical Pharmacology of Utrecht University.

### Questionnaire

Based on monitoring recommendations in various guidelines, a questionnaire was designed to assess how health care professionals prescribing lithium to patients with bipolar disorder report to monitor these patients. The primary researcher (MN) assessed all monitoring instructions in four guidelines (Kupka et al. 2015; National Institute for Health and Care Excellence 2014; Ng et al. 2009; Grunze et al. 2013), to explore relevant monitoring parameters and advised frequencies of monitoring: the Dutch Multidisciplinary guideline (Kupka et al. 2015), the International Society for Bipolar Disorders guidelines (Ng et al. 2009) and the World Federation of Societies of Biological Psychiatry guidelines (Grunze et al. 2013), and the guideline of the National Institute for Health and Care Excellence (2014). Actual utilization of guidelines was assessed, including explicatory reasons. In addition, we included questions on local monitoring systems. Most questions were multiple choice with an option offered for comments at the end, if relevant. The study group then composed a questionnaire in LimeSurvey version 2.5, an online accessible survey application. The survey was tested by eleven Dutch experts in the field of lithium treatment (Additional file 2: Appendix S2) to optimize content on appropriateness, relevance, and time to complete the survey, and it was adjusted accordingly.

The final questionnaire consisted of 41 questions divided into four parts. First, target lithium serum levels and frequency of monitoring were assessed. Second, monitoring frequency of physical and laboratory parameters was assessed. Third, to identify factors contributing to monitoring, reasons to monitor or not to monitor, and use of guidelines or institutional protocols were investigated. Fourth, personal characteristics of the respondent including age, gender, experience with prescribing lithium, and country of residence were collected. A condensed version of the questionnaire can be found in Table 1. The full questionnaire can be found in Additional file 3: Appendix 3.

**Table 1:** Aspects covered in the questionnaire on monitoring of patients using lithium

Aspect	Item number	Items of question
Lithium serum level monitoring	1–8	Target values for: acute manic episode in adults (18–60 years old), maintenance treatment in adults, acute manic episode in elderly patients (> 60 years old), maintenance treatment in elderly Frequency of monitoring lithium serum levels during the first month, months 2–6 and per year
Monitoring of physical and laboratory parameters	9–25	Assessment of the following monitoring parameters before, during the first 6 months and during maintenance treatment Physical parameters: bodyweight, body mass index (BMI), blood pressure, pulse, waist circumference, electrocardiogram (ECG), pregnancy test, 24 h urine examination Laboratory parameters: creatinine, urea, albumin, glomerular filtration rate (GFR), thyroid stimulating hormone (TSH), parathyroid hormone (PTH), thyroxine (T4), alanine aminotransferase (ALAT), aspartate aminotransferase (ASAT), bilirubin, sodium, potassium, calcium, full blood count, leukocytes, leukocyte differentiation, total cholesterol, high density lipoprotein (HDL), low density lipoprotein (LDL), very low density lipoprotein (vLDL), triglycerides, fasting glucose
Local system for ensuring monitoring	26–35	Reasons to and not to monitor, assessment usage of guidelines or institutional protocols, responsible health care professional for monitoring, usage of laboratory protocols, method of inviting patients for monitoring
Background information	36–41	Gender, age, profession, institution of employment, years of lithium prescribing, country

### Analysis

Responses were analyzed using the Statistical Package for Social Sciences Version 24.0 for Windows (SPSS 24.0; SPSS Inc, Chicago, IL). Data were analyzed using descriptive statistics. Target lithium serum levels were presented with the use of boxplot statistics using Tukey’s Hinges (Tukey 1977).

## Results

### Study sample

A total of 117 respondents completed the survey. The majority of respondents were employed as psychiatrists (91%), followed by nurse practitioners (3%), psychiatric residents (5%), or as general practitioner (1%). Twenty percent of the respondents were self-employed. The duration of experience with prescribing lithium had a median of 22 years [interquartile range (IQR) 10–33]. Two-third (66%) of the respondents were male; the median age was 53 years (IQR 41–60).

**Fig. 1:**
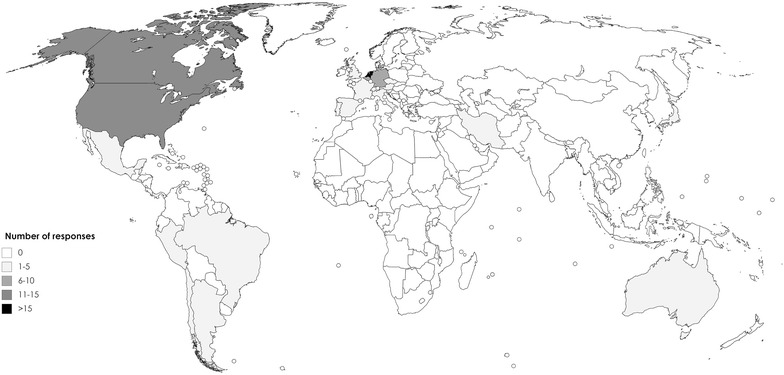
Demographic presentation of responses

Responses were collected from health care professionals residing in twenty-four different countries. Countries where prescribers were employed were diverse, with 63% of the respondents employed in Europe, 22% in North America, 9% in South America, 3% in Australia, 2% in Asia and 1% in Africa (Fig. 1).

### Lithium serum level and frequency of monitoring

**Fig. 2:**
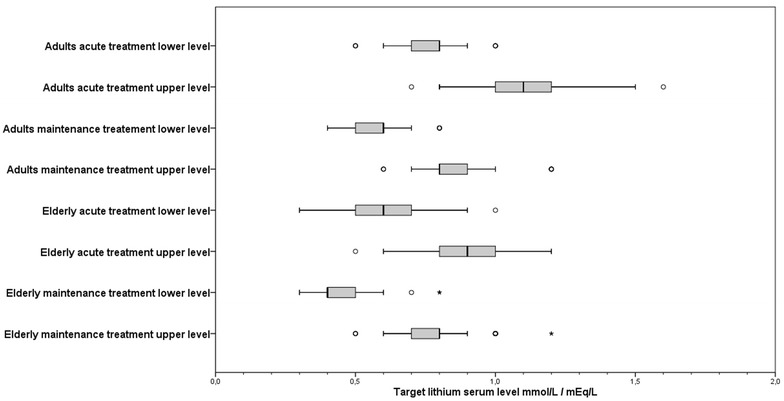
Target lithium serum levels for both adults and elderly patients (> 60 years) during acute and maintenance treatment

In Fig. 2, target values for minimum and maximum lithium plasma levels the respondents reported to pursue are presented for acute and maintenance treatment in adults and elderly patients.

For adults, the median lower target level for acute treatment was 0.8 mmol/L (IQR 0.1; range 0.5–1.0). The median for the upper level was 1.1 mmol/L (IQR 0.2; range 0.7–1.6). For maintenance treatment the median target serum level for adults was 0.6 mmol/L (IQR 0.1; range 0.4–0.8); with a median upper level of 0.8 mmol/L (IQR 0.1; range 0.5–1.2).

For elderly, the median target range for serum levels was 0.6 mmol/L (IQR 0.2; range 0.3–1.0), with 0.9 mmol/L (IQR 0.2; range 0.5–1.2) as upper level for acute treatment. During maintenance treatment the median lower level was 0.4 mmol/L (IQR 0.1; range 0.3–0.8); with 0.8 mmol/L (IQR 0.2; range 0.6–1.2) as upper level. Spread in target ranges for both adults and elderly patients during acute treatment was relatively larger compared to maintenance treatment.

All respondents monitored lithium serum levels during treatment, although there was variability in frequency. In the initial phase of lithium treatment, most respondents monitored lithium serum levels one to three times in the first month and between one and three times during months 2–6. During maintenance treatment half of the respondents monitored one to three times a year; others four or more times a year (44%) (Table 2). Many had difficulties explaining how often they monitored the lithium serum level during the first month, as they monitored lithium serum levels until they reached the target level. Thirteen respondents commented to monitor 5–7 days after every dose change.

**Table 2:** Frequency of monitoring lithium serum levels

Frequency of monitoring	Never	1–3×	≥ 4×
First month	0	75 (64%)	42 (36%)
Months 2–6	0	84 (72%)	33 (28%)
Per year (during maintenance treatment)	0	65 (56%)	52 (44%)

### Monitoring of physical and laboratory parameters

All responders monitored at least one physical or laboratory parameter. Responses of monitoring of physical and laboratory monitoring are shown in Table 3.

**Table 3:** Monitoring of physical and laboratory parameters

Parameter	Monitoring before start of treatment	Frequency of monitoring during the first 6 months of treatment			Frequency of monitoring per year during maintenance treatment			
		Never	1–3×	≥ 4×	Never	Once	2–3×	≥ 4×
Physical parameters								
Physical parameters (at least one parameter)	104 (88%)	17 (14%)	*76 (64%)*	24 (21%)	12 (10%)	*58 (49%)*	28 (24%)	19 (16%)
Body weight	92 (79%)	32 (27%)	*70 (60%)*	15 (13%)	20 (17%)	*58 (50%)*	25 (21%)	14 (12%)
Blood pressure	88 (75%)	35 (30%)	*63 (54%)*	19 (16%)	28 (24%)	*51 (44%)*	24 (21%)	14 (12%)
Pulse	79 (68%)	43 (37%)	*56 (48%)*	18 (15%)	38 (32%)	*48 (41%)*	19 (16%)	12 (10%)
Body mass index (BMI)	63 (54%)	*54 (46%)*	*54 (46%)*	9 (8%)	45 (38%)	*47 (40%)*	16 (14%)	9 (8%)
Electrocardiogram (ECG)	51 (44%)	*66 (56%)*	46 (39%)	5 (4%)	*71 (61%)*	31 (27%)	12 (10%)	3 (3%)
Waist circumference	45 (38%)	*72 (62%)*	42 (36%)	3 (3%)	*65 (56%)*	38 (32%)	9 (8%)	5 (4%)
Pregnancy test	40 (34%)	*90 (77%)*	24 (21%)	3 (3%)	*102 (87%)*	7 (6%)	6 (5%)	2 (2%)
24 h urine examination	23 (20%)	*93 (79%)*	21 (18%)	3 (3%)	*88 (75%)*	23 (20%)	2 (2%)	4 (3%)
Renal function								
Renal function (at least one parameter)	116 (99%)	3 (3%)	*88 (75%)*	26 (22%)	0	25 (21%)	*60 (51%)*	32 (27%)
Creatinine	116 (99%)	3 (3%)	*88 (75%)*	26 (22%)	0	26 (22%)	*59 (50%)*	32 (27%)
Urea	101 (86%)	21 (18%)	*78 (67%)*	18 (15%)	18 (15%)	22 (19%)	*48 (41%)*	29 (25%)
Glomerular filtration rate (GFR)	100 (85%)	19 (16%)	*76 (65%)*	22 (19%)	13 (11%)	22 (19%)	*52 (44%)*	30 (26%)
Albumin	66 (56%)	46 (39%)	*60 (51%)*	11 (9%)	*42 (36%)*	21 (18%)	34 (29%)	20 (17%)
Thyroid function								
Thyroid function (at least one parameter)	115 (98%)	2 (2%)	*104 (89%)*	11 (9%)	0	43 (36%)	*54 (46%)*	20 (17%)
Thyroid stimulating hormone (TSH)	115 (98%)	2 (2%)	*104 (89%)*	11 (9%)	1 (1%)	42 (36%)	*54 (46%)*	20 (17%)
Thyroxine (T4)	82 (70%)	30 (26%)	*80 (68%)*	7 (6%)	28 (24%)	33 (28%)	*41 (35%)*	15 (13%)
Parathyroid function								
Parathyroid hormone (PTH)	32 (27%)	*79 (68%)*	35 (30%)	3 (3%)	*72 (62%)*	22 (19%)	17 (15%)	6 (5%)
Electrolytes								
Electrolytes (at least one parameter)	107 (91%)	10 (9%)	*86 (73%)*	21 (18%)	8 (7%)	34 (29%)	*49 (42%)*	26 (22%)
Sodium	103 (88%)	17 (15%)	*80 (68%)*	20 (17%)	14 (12%)	32 (27%)	*46 (39%)*	25 (21%)
Potassium	101 (86%)	18 (15%)	*79 (68%)*	20 (17%)	15 (13%)	32 (27%)	*45 (38%)*	25 (21%)
Calcium	94 (80%)	21 (18%)	*81 (69%)*	15 (13%)	19 (16%)	35 (30%)	*44 (38%)*	19 (16%)
Hematological parameters								
Hematological parameters (at least one parameter)	106 (90%)	26 (22%)	*79 (67%)*	12 (10%)	24 (21%)	*51 (44%)*	29 (25%)	13 (11%)
Full blood count	102 (87%)	32 (27%)	*73 (62%)*	12 (10%)	30 (26%)	*47 (40%)*	29 (25%)	11 (9%)
Leukocytes	101 (86%)	30 (26%)	*75 (64%)*	12 (10%)	27 (23%)	*50 (43%)*	27 (23%)	13 (11%)
Leukocyte differentiation	87 (74%)	40 (34%)	*67 (57%)*	10 (9%)	38 (32%)	*45 (38%)*	25 (21%)	9 (8%)
Lipid profile								
Lipid profile (at least one parameter)	73 (62%)	*57 (49%)*	55 (47%)	5 (4%)	42 (36%)	*53 (45%)*	18 (15%)	4 (3%)
Triglycerides	71 (61%)	*58 (50%)*	54 (46%)	5 (4%)	43 (37%)	*52 (44%)*	18 (15%)	4 (3%)
Total cholesterol	71 (61%)	*59 (50%)*	53 (45%)	5 (4%)	44 (38%)	*52 (44%)*	17 (15%)	4 (3%)
High density lipoprotein (HDL)	69 (59%)	*60 (51%)*	53 (45%)	4 (3%)	45 (38%)	*51 (44%)*	18 (15%)	3 (3%)
Low density lipoprotein (LDL)	67 (57%)	*63 (54%)*	50 (43%)	4 (3%)	48 (41%)	*49 (42%)*	17 (15%)	3 (3%)
Very low density lipoprotein (vLDL)	35 (30%)	*84 (72%)*	32 (27%)	1 (1%)	*71 (61%)*	35 (30%)	11 (9%)	0
Hepatic function								
Hepatic function (at least one parameter)	68 (58%)	*61 (52%)*	48 (41%)	8 (7%)	*54 (46%)*	37 (32%)	20 (17%)	6 (5%)
Alanine aminotransferase (ALAT)	68 (58%)	*62 (53%)*	47 (40%)	8 (7%)	*55 (47%)*	37 (32%)	19 (16%)	6 (5%)
Aspartate aminotransferase (ASAT)	66 (56%)	*64 (55%)*	46 (39%)	7 (6%)	*55 (47%)*	37 (32%)	19 (16%)	6 (5%)
Bilirubin	34 (29%)	*79 (68%)*	33 (28%)	5 (4%)	*71 (61%)*	27 (23%)	15 (13%)	3 (3%)
Other laboratory parameters								
Fasting glucose	79 (67%)	48 (41%)	*63 (54%)*	6 (5%)	35 (30%)	*56 (48%)*	20 (17%)	6 (5%)

Italicized cells represent most reported answers after initiation of lithium treatment

Most respondents monitored bodyweight, blood pressure, pulse, and body mass index. Fewer respondents made electrocardiograms, monitored waist circumference, performed pregnancy tests, or 24 h urine examinations. Renal function and thyroid function were monitored by almost all respondents before start and during maintenance treatment. Primarily creatinine, thyroid stimulating hormone (TSH), urea, and glomerular filtration rate were measured. PTH was considerably less often monitored, sometimes solely in case of a deviating calcium level. A few mentioned to measure anti-thyroid autoantibodies (n = 6), mostly in case of abnormal TSH. Electrolytes and hematological parameters were generally determined before start and often repeated during maintenance treatment. Slightly more than half of the respondents monitored a lipid profile and hepatic function before start. Most of them never monitored hepatic function after start, while lipids were monitoring more often once a year during maintenance treatment. Fasting glucose was monitored by about two-thirds of the respondents before start, and about half of the respondents monitored fasting glucose one to three times during the first 6 months and thereafter once a year during maintenance treatment.

Some respondents mentioned to monitor the following additional parameters: alkaline phosphatase (ALP) (n = 1), CRP (n = 1), cysteine (n = 1), EEG (n = 1), erythrocyte sedimentation rate (n = 1), folate (n = 2), gamma-glutamyltransferase (GGT) (n = 1), glycated hemoglobin (HbA1c) (n = 1), serum level of antipsychotic agents (n = 1), urine osmolality (n = 1) vitamin D (n = 4), physical examination (n = 1), vitamin B1 or vitamin B12 (n = 2).

### Reasons for monitoring

The main reason to monitor patients using lithium was because of safety or adverse effects (99%). Respondents monitored additionally to aid in dose adjustment or optimization (94%), in case of possible lithium toxicity (85%), during start, stop, or dose-adjustment of interacting medication (67%), to verify therapeutic level, especially in case of insufficient efficacy (64%), due to co-morbidities of the patient (63%), and because it is recommended in guidelines (59%). Other mentioned reasons that were to assess compliance (3%), to monitor patients during pregnancy (1%) or in case of a new episode (1%), or if a medication brand changed (1%).

Reasons not to monitor patients during lithium treatment were a lack of resources to monitor (4%), non-cooperative patients (4%), if a guideline did not obligate monitoring (3%), if monitoring was unnecessary in the opinion of the respondent (3%), if an institutional protocol did not require monitoring (2%), or unawareness of the necessity of monitoring (2%). Still, 90% of the respondents reported they always monitored their patients. One psychiatrist commented that there are no good reasons *not* to monitor a patient on lithium.

### Use of guidelines or institutional protocols for monitoring

The monitoring policy of 74% of the respondents was based upon a guideline or institutional protocol. A minority of respondents said not to use guidelines because personal experience and practice were more valuable to them (9%). A number of respondents thought guidelines were not explicit enough (7%) or the institutional protocol or Summary of Product Characteristics (SmPC) they used was overlapping (4%). Another reason not to use guidelines was a difficulty to apply them in daily clinical practice (4%), including a lack of resources in order to monitor (2%). Four psychiatrists (3%) reported the institutional protocol/SmPC they used was sufficient. Alternative reasons not to use guidelines were difficulties to apply guidelines to their patient population (3%) or an unawareness of the existence of such guidelines (2%). Additionally, respondents mentioned to perform monitoring based on individual patient characteristics (2%) or to experience a difficulty to choose between guidelines (1%). Sometimes guidelines were found to be excessive (1%) or they used their own reading of literature (1%).

### Local systems for ensuring monitoring patients treated with lithium

The majority of respondents reported to be personally responsible for monitoring (91%). The health care professional responsible for monitoring was a psychiatrist (83%), psychiatric resident (3%), nurse practitioner (3%), or the head/clinic nurse (3%). A shared responsibility was mentioned by some respondents (3%). In a few responses, a general practitioner (2%), family physician (1%) or the doctor who continued treatment after discharge (1%) was responsible for monitoring.

Among the respondents, 74% had to request each parameter separately. For monitoring a combination of parameters during lithium treatment, 25% could make use of an existing laboratory protocol. For some health care professionals, the system for ensuring monitoring was organized in a way that patients were automatically invited by a laboratory or physician for determination of monitoring parameters (26%).

## Discussion

We surveyed prescribers of lithium on monitoring of patients using lithium for bipolar disorder. All prescribers reported to monitor lithium serum levels on a regular basis, with varying intervals and target levels. Target levels for maintenance treatment were mostly consistent with target ranges in guidelines for bipolar disorders (Malhi et al. 2017). Target levels for acute treatment were more variable, which is in accordance with the variability among guidelines (Malhi et al. 2017).

Almost all health care professionals monitored thyroid and renal function before start, and all during maintenance treatment, indicating a higher priority was given to these parameters compared to other parameters. This does not mean that all respondents gave less priority to other monitoring physical or laboratory parameters. Some may consider other parameters equally important, but our results show that they reported to monitor at least renal and thyroid function. Respondents may not monitor these parameters in *all* patients using lithium in clinical practice. From studies in The Netherlands and the UK it is known that monitoring rates may be much lower than the health care professionals reported in our survey; showing that 8–22% of patients did not have any recorded test for renal or thyroid function (van de Beek et al. 2010; Paton et al. 2013). A difference between reported and actual monitoring in clinical practice can be a consequence of inadequate adherence to monitoring by patients (Collins et al. 2010). It is possible that respondents despite anonymity may have given socially desirable answers to our survey. If patients using lithium are not adequately monitored, ADEs may not be identified in time. On the other hand, excessive monitoring may have a negative impact on health care costs and provide an extra burden to patients and may even lead to a reduced treatment adherence (Tran et al. 2015).

Reported monitoring was more variable for other physical and laboratory parameters than thyroid and renal function. It would be of interest to assess reasons of health care professionals to monitor or not to monitor these parameters. Variability in monitoring between respondents can be a consequence of a lack of resources, difference in education, type of patient population, a different opinion on the need for monitoring or differences in recommendations in guidelines (Zivanovic 2017). The number of responses was not sufficient to compare responses between subgroups such as countries, type of health care professional or to determine guideline adherence.

Overall, respondents reported to use guidelines or institutional protocols for monitoring. Guidelines differ with respect to their recommendations, and studies have shown that there are discrepancies between guideline recommendations and actual monitoring in clinical practice (Malhi et al. 2017; van de Beek et al. 2010; Paton et al. 2013; Minay et al. 2013). Reasons to use or not to use guidelines were assessed in our survey. In line with previous research, several respondents reported not to follow guidelines because in their opinion they did not apply to their patient population (Perlis 2007). Guidelines are mainly based on literature such as randomized controlled trials that may not include specific patient populations, for instance children or older patients (Zarin et al. 2005). In our survey, health care professionals mentioned personal experience and practice to be the main reasons for not following a guideline. A lack of resources and non-cooperative patients could further complicate monitoring. Not all laboratories are equipped to perform all laboratory tests (Guo et al. 2013) and for prescribers of lithium not all monitoring facilities may be easily accessible, for example for making an electrocardiogram.

In the majority of cases the prescriber of lithium, generally a psychiatrist, was responsible for monitoring. The health care system should be organized in a way that continuity of monitoring is warranted. For instance, in the Dutch guideline, the prescriber is appointed to be responsible for monitoring (Kupka et al. 2015), but another health care professional can have a coordinating function. Among respondents, a small group reported that patients were automatically invited by a laboratory or physician for monitoring. Most responders had to request each monitoring parameter separately, which may increase the workload and demands awareness of health care professionals. A method to order all monitoring parameters after a prescription has been described, known as a corollary order (Wright and Sittig 2006). In this way, an order set of monitoring parameters could be developed to be used when a health care professional prescribes lithium. Clinical decision systems could be programmed in a way that all relevant monitoring parameters are automatically ordered after a lithium prescription.

### Strengths and limitations

An advantage of using a survey is that we could assess anonymized reported monitoring and use of guidelines. Our results help to understand why prescribers of lithium monitor patients and what their main reasons are not to monitor patients according to guideline recommendations or even not at all. By using an international online survey we were able to include respondents from 24 countries worldwide.

A limitation is that the response was not sufficient to compare individual countries. The limited response could have been a result of a limited amount of time or a lack of interest in the subject. Since the survey was distributed via various networks, we do not know how many professionals actually have been reached. Prescribers who did participate in the survey, may have been a selection of prescribers who are more interested in the subject. The survey was sent to health care professionals who were members of professional health care organizations, including those with a specific focus on bipolar disorder. It is expected that they are better informed on lithium monitoring as some of them may have a special interest in lithium treatment. Respondents were relatively experienced, with a median of 22 years of prescribing lithium. Therefore, responses may not reflect monitoring in general clinical practice internationally.

## Conclusions

Adequate monitoring of patients using lithium is needed for optimal dosing and early identification of patients with (potential) ADEs. In our survey, health care professionals prescribing lithium report to monitor lithium serum levels, renal and thyroid function on a regular basis. However, there was considerable variation in other monitoring parameters. Our results help to understand why prescribers of lithium monitor patients and what their main reasons are not to monitor patients. In future, it will be of interest to assess how prescribers respond to deviating parameters.

## Additional files

**Additional file 1: Appendix S1.** Networks of professional organizations.

**Additional file 2: Appendix S2.** List of Dutch lithium experts who contributed to the pilot study.

**Additional file 3: Appendix S3.** Lithium survey.

## Abbreviations

ADE: adverse drug event
ALP: alkaline phosphatase
eGFR: estimated glomerular filtration rate
GGT: gamma-glutamyltransferase
HbA1c: glycated hemoglobin
IGSLi: International Group for The Study of Lithium Treated Patients
IQR: interquartile range
PTH: parathyroid hormone
SmPC: Summary of Product Characteristics
TSH: thyroid stimulating hormone
