# Supplementary material for: Monitoring of patients treated with lithium for bipolar disorder: an international survey
Source: Int J Bipolar Disord. 2018 Apr 14;6:12. doi: 10.1186/s40345-018-0120-1 (PMC6161983; doi:10.1186/s40345-018-0120-1)
Supplement: Supplementary file 1 — Additional file 1: Appendix S1. Networks of professional organizations. [file 40345_2018_120_MOESM1_ESM.docx]

**Appendix S1 – Networks of professional organizations**

| **Network** |
| --- |
| International Group for The Study of Lithium Treated Patients (IGSLI) |
| Dutch Foundation for Bipolar Disorders |
| International Society for Bipolar Disorders (ISBD) |
| Belgian College of Neurological and Biological Psychiatry (BCNBP) |
| Arbeitsgemeinschaft für Neuropsychopharmakologie und Pharmakopsychiatrie (AGNP) |
| Altrecht Institute for Mental Health Care, The Netherlands |
| Rivierduinen, Institute for Mental Health Care, The Netherlands |
| Eleos, Mental Health Care, The Netherlands |
| Antes, Delta Psychiatric Center, The Netherlands |
