# Supplementary material for: Monitoring of patients treated with lithium for bipolar disorder: an international survey
Source: Int J Bipolar Disord. 2018 Apr 14;6:12. doi: 10.1186/s40345-018-0120-1 (PMC6161983; doi:10.1186/s40345-018-0120-1)
Supplement: Supplementary file 2 — Additional file 2: Appendix S2. List of Dutch lithium experts who contributed to the pilot study. [file 40345_2018_120_MOESM2_ESM.docx]

**Appendix S2 – List of Dutch lithium experts who contributed to the pilot study**

A. Dols; A.C.G. Egberts; E.R. Heerdink; R. Hoekstra; R.W. Kupka; E.J.M. van Melick; M. Nederlof; J. Renes; E.J. Regeer; L.J. Stoker; I. Wilting
