# Supplementary material for: Monitoring of patients treated with lithium for bipolar disorder: an international survey
Source: Int J Bipolar Disord. 2018 Apr 14;6:12. doi: 10.1186/s40345-018-0120-1 (PMC6161983; doi:10.1186/s40345-018-0120-1)
Supplement: Supplementary file 3 — Additional file 3: Appendix S3. Lithium survey. [file 40345_2018_120_MOESM3_ESM.docx]

**Appendix S3 – Lithium survey**

**Worldwide characteristics of lithium monitoring in guidelines and in clinical practice**

Lithium is used for the treatment of bipolar disorder. It is a drug with a narrow therapeutic index and can cause severe side effects. National and international guidelines advise the monitoring of lithium serum levels and of physical and laboratory parameters. However, it is unclear how and when monitoring takes place in clinical practice.

The aim of this questionnaire is to establish how monitoring during lithium therapy takes place internationally. We will compare the results of multiple countries and additionally assess guidelines on monitoring during lithium therapy.

This survey will address the topics of lithium serum level monitoring, monitoring of physical and laboratory parameters and the habits of and reasons for monitoring during lithium therapy in bipolar disorder.

This survey is aimed at the treatment and monitoring of adults (age ≥ 18 years) **with bipolar disorder**.

The answers will be processed anonymously. The survey will take approximately 15 minutes.

The researchgroup:
M. Nederlof, Utrecht University
R.W. Kupka, VU University Medical Center
A.M. Braam, Utrecht University
L.J. Stoker, Utrecht University
R. Hoekstra, Delta Psychiatric Centre
T.C.G. Egberts, University Medical Center Utrecht, Utrecht University
E.R. Heerdink, Utrecht University

There are 41 questions in this survey

**Part 1: Lithium serum level monitoring**

1. How do you usually divide the lithium dosage during the day?

- Once a day in the morning
- Once a day in the evening
- Twice a day
- Other

2. What reference values for the lithium serum level do you most commonly aim for **during an acute manic episode in adult patients** (from 18 to 60 years old)?

Lower limit (mmol/L or mEq/L) Upper limit (mmol/L or mEq/L)

Between 0,1 – 2,0 (steps 0,1) Between 0,1 – 2,0 (steps 0,1)

3. What references values for the lithum serum level do you most commonly aim for during **maintenance** **treatment in adult patients** (from 18 to 60 years old)?

Lower limit (mmol/L or mEq/L) Upper limit (mmol/L or mEq/L)

Between 0,1 – 2,0 (steps 0,1) Between 0,1 – 2,0 (steps 0,1)

4. What references values for the lithium serum level do you most commonly aim for **during an acute manic episode in elderly patients** (>60 years old)?

Lower limit (mmol/L or mEq/L) Upper limit (mmol/L or mEq/L)

Between 0,1 – 2,0 (steps 0,1) Between 0,1 – 2,0 (steps 0,1)

5. What reference values for the lithium serum level do you most commonly aim for during **maintenance** **therapy in elderly patients** (>60 years old)?

Lower limit (mmol/L or mEq/L) Upper limit (mmol/L or mEq/L)

Between 0,1 – 2,0 (steps 0,1) Between 0,1 – 2,0 (steps 0,1)

6. How often do you monitor the lithium serum level **during the first month** of treatment?

- Never
- Once
- Twice (every other week)
- Four times (every week)
- Eight times (twice a week)
- Every other day
- Every day

Comment:

7. How often do you usually monitor the lithium serum level in total **during months 2-6**?

- Never
- 1-3 x
- 4-6 x
- 7-9 x
- 10-12 x
- >12 x

Comment:

8. When you continue lithium therapy, how often do you monitor the lithium serum level **per year**?

Never

- 1 x
- 2 x
- 3 x
- 4 x
- >4 x

Comment:

**Part 2: Monitoring of physical and laboratory parameters**

9. Please indicate if you monitor the following physical parameters **before** start of therapy

*Parameters Yes No*

Bodyweight ○ ○

Body mass index (BMI) ○ ○

Blood pressure ○ ○

Pulse ○ ○

Waist circumference ○ ○

Electrocardiogram (ECG) ○ ○

Pregnancy test ○ ○

24h urine examination ○ ○

10. Please indicate if you monitor the following parameters of renal function **before** start of therapy

*Parameter Yes No*

Creatinine ○ ○

Urea ○ ○

Albumin ○ ○

Glomeralur filtration rate (GFR) ○ ○

11. Please indicate if you monitor the following parameters of thyroid function **before** start of therapy

*Parameter Yes No*

Thyroid stimulating hormone (TSH) ○ ○

Parathyroid hormone (PTH) ○ ○

Thyroxine (T4) ○ ○

12. Please indicate if you monitor the following parameters of hepatic function **before** start of therapy

*Parameter Yes No*

Alanine aminotransferase (ALAT) ○ ○

Aspartate aminotransferase (ASAT) ○ ○

Bilirubin ○ ○

13. Please indicate if you monitor the following electrolytes **before** start of therapy

*Parameter Yes No*

Sodium ○ ○

Potassium ○ ○

Calcium ○ ○

14. Please indicate if you monitor the following haematological parameters **before** start of therapy

*Parameters Yes No*

Full blood count ○ ○

Leukocytes ○ ○

Leukocyte differentiation ○ ○

15. Please indicate if you monitor the following components of lipid and metabolic profile **before** start of therapy

*Parameter Yes No*

Total cholesterol ○ ○

High density lipoprotein (HDL) ○ ○

Low density lipoprotein (LDL) ○ ○

Very low density lipoprotein (vLDL) ○ ○

Triglycerides ○ ○

Fasting glucose ○ ○

16. Do you monitor additional parameters?

- No

- Yes, namely

17. Explanation, if needed:

18. Pease indicate if and how often you monitor the following physical parameters during **the first 6 months of therapy** and during **maintenance therapy**

*First 6 months of therapy Maintenance therapy (per year)*

Parameter Never 1-3x 4-6x 7-9x 10-12x >12x Never 1x 2x 3x 4x >4x

Bodyweight ○ ○ ○ ○ ○ ○ ○ ○ ○ ○ ○ ○

Body mass index (BMI) ○ ○ ○ ○ ○ ○ ○ ○ ○ ○ ○ ○

Blood pressure ○ ○ ○ ○ ○ ○ ○ ○ ○ ○ ○ ○ Pulse ○ ○ ○ ○ ○ ○ ○ ○ ○ ○ ○ ○

Waist circumference ○ ○ ○ ○ ○ ○ ○ ○ ○ ○ ○ ○ Electrocardiogram (ECG) ○ ○ ○ ○ ○ ○ ○ ○ ○ ○ ○ ○

Pregnancy test ○ ○ ○ ○ ○ ○ ○ ○ ○ ○ ○ ○

24h urine examination ○ ○ ○ ○ ○ ○ ○ ○ ○ ○ ○ ○

19. Pease indicate if and how often you monitor the following parameters or renal function during **the first 6 months of therapy** and during **maintenance therapy**

*First 6 months of therapy Maintenance therapy (per year)*

Parameter Never 1-3x 4-6x 7-9x 10-12x >12x Never 1x 2x 3x 4x >4x

Creatinine ○ ○ ○ ○ ○ ○ ○ ○ ○ ○ ○ ○

Urea ○ ○ ○ ○ ○ ○ ○ ○ ○ ○ ○ ○

Albumin ○ ○ ○ ○ ○ ○ ○ ○ ○ ○ ○ ○ Glomerular filtration rate ○ ○ ○ ○ ○ ○ ○ ○ ○ ○ ○ ○

20. Pease indicate if and how often you monitor the following parameters or thyroid function during **the first 6 months of therapy** and during **maintenance therapy**

*First 6 months of therapy Maintenance therapy (per year)*

Parameter Never 1-3x 4-6x 7-9x 10-12x >12x Never 1x 2x 3x 4x >4x

Thyroid stimulating ○ ○ ○ ○ ○ ○ ○ ○ ○ ○ ○ ○

hormone (TSH)

Parathyroid hormone ○ ○ ○ ○ ○ ○ ○ ○ ○ ○ ○ ○

(PTH)

Thyroxine (T4) ○ ○ ○ ○ ○ ○ ○ ○ ○ ○ ○ ○

21. Pease indicate if and how often you monitor the following parameters or hepatic function during **the first 6 months of therapy** and during **maintenance therapy**

*First 6 months of therapy Maintenance therapy (per year)*

Parameter Never 1-3x 4-6x 7-9x 10-12x >12x Never 1x 2x 3x 4x >4x

Alanine aminotransferase ○ ○ ○ ○ ○ ○ ○ ○ ○ ○ ○ ○

(ALAT)

Aspartate aminotrans- ○ ○ ○ ○ ○ ○ ○ ○ ○ ○ ○ ○

ferase (ASAT)

Bilirubin ○ ○ ○ ○ ○ ○ ○ ○ ○ ○ ○ ○

22. Pease indicate if and how often you monitor the following electrolytes during **the first 6 months of therapy** and during **maintenance therapy**

*First 6 months of therapy Maintenance therapy (per year)*

Parameter Never 1-3x 4-6x 7-9x 10-12x >12x Never 1x 2x 3x 4x >4x

Sodium ○ ○ ○ ○ ○ ○ ○ ○ ○ ○ ○ ○

Potassium ○ ○ ○ ○ ○ ○ ○ ○ ○ ○ ○ ○

Calcium ○ ○ ○ ○ ○ ○ ○ ○ ○ ○ ○ ○

23. Pease indicate if and how often you monitor the following haematological parameters during **the first 6 months of therapy** and during **maintenance therapy**

*First 6 months of therapy Maintenance therapy (per year)*

Parameter Never 1-3x 4-6x 7-9x 10-12x >12x Never 1x 2x 3x 4x >4x

Full blood count ○ ○ ○ ○ ○ ○ ○ ○ ○ ○ ○ ○

Leukocytes ○ ○ ○ ○ ○ ○ ○ ○ ○ ○ ○ ○

Leukocyte differentiation ○ ○ ○ ○ ○ ○ ○ ○ ○ ○ ○ ○

24. Pease indicate if and how often you monitor the following components of lipid and metabolic profile during **the first 6 months of therapy** and during **maintenance therapy**

*First 6 months of therapy Maintenance therapy (per year)*

Parameter Never 1-3x 4-6x 7-9x 10-12x >12x Never 1x 2x 3x 4x >4x

Total cholesterol ○ ○ ○ ○ ○ ○ ○ ○ ○ ○ ○ ○

High density lipoprotein ○ ○ ○ ○ ○ ○ ○ ○ ○ ○ ○ ○

(HDL)

Low density lipoprotein ○ ○ ○ ○ ○ ○ ○ ○ ○ ○ ○ ○

(LDL)

Very low density ○ ○ ○ ○ ○ ○ ○ ○ ○ ○ ○ ○

lipoprotein (vLDL)

Triglycerides ○ ○ ○ ○ ○ ○ ○ ○ ○ ○ ○ ○ Fasting glucose ○ ○ ○ ○ ○ ○ ○ ○ ○ ○ ○ ○

25. Explanation, if needed:

**Part 3: System of monitoring**

26. What are reasons to monitor during lithium therapy (both lithium serum level and other parameters)? (Please choose all that apply)

- Not applicable, I never start monitoring
- Safety / side-effects
- Efficacy
- Dose-adjustment / optimization
- Start/stop/dose-adjustment of interacting medication
- Recommendations in guidelines
- Co-morbidities of the patient
- Suspicion of lithium toxicity
- Other: .

27. What are reasons **NOT** to monitor during lithium therapy (both lithium serum level and other parameters)?

(Please choose all that apply)

- Not applicable, I always start monitoring
- It is in my belief unnecessary
- The guideline doesn’t oblige me to do it
- The SmPC doesn’t oblige me to do it
- My institutional protocol doesn’t oblige me to do it
- I’m not aware of the necessity of monitoring
- The patient is not co-operative
- I don’t have the resources to monitor
- Other: .

28. Is your monitoring policy based on a guideline or institutional protocol?

- Yes
- No

29. Which guideline / institutional protocol?

Please write your answer here:

30. Please upload the institutional protocol or guideline you use in lithium monitoring. If you only have a link to the guideline or protocol you can send this in the next question.

Kindly attach the aforementioned documents along with the survey

31. Please send the link of the guideline or institutional protocol you use in lithium monitoring

Please write your answer here:

32. What are reasons NOT to use the guidelines during lithium therapy?

(Please choose all that apply)

- Not applicable, I always use the guidelines
- I’m not aware of the existence of such guidelines
- The institutional protocol/ SmPC I use is sufficient
- The institutional protocol/ SmPC I use is overlapping
- Personal experience and practices are more valuable than guidelines
- The guidelines are difficult to apply in daily practice
- The guidelines are not applicable to my patient population
- The guidelines are not explicit enough
- Other: .

33. Who is responsible for the monitoring?

(If you choose ‘Other:’ please also specify your choice in the accompanying text field.)

- I am
- Other: .

34. How is the monitoring requested?

(If you choose ‘Other:’ please also specify your choice in the accompanying text field.)

- I have to request every parameter separately
- There is a laboratory protocol for monitoring all parameters during lithium therapy
- Other: .

35. Is the patient automatically invited by a laboratory or physician for determination of monitoring parameters by use of a protocol?

- Yes
- No

**Part 4: Background information**

36. What is your gender?

- Male
- Female

37. What is your age?

(Answer must be between 18 and 100).

Please write your answer here:

years

38. What is your profession?

- Psychiatrist
- Internist
- General practitioner
- Nurse practitioner
- Other: .

39. At what kind of institution are you employed? *

(Please choose all that apply)

- A general hospital
- A public hospital
- I’m a self-employed psychiatrist/general practitioner
- Other: .

* This question was removed from the analysis, because the difference between a general and public hospital was unclear.

40. How many years have you been prescribing lithium?

Between 0 – 50 (steps 1)

41. In which country are you currently working?

(Choose one of the following)

Thank you for filling out our questionnaire!

If you have any questions or remarks about the questionnaire or research you can contact the researchgroup at:
M.Nederlof@uu.nl

or

Utrecht University
Department of Pharmaceutical Sciences
Division of Pharmacoepidemiology and Clinical Pharmacology
P.O. Box 80082
3508 TB Utrecht
The Netherlands
Phone : +31 (0)30 253 7324
10-04-2017 – 13:05

Submit your survey.
Thank you for completing this survey.
